# Poor platelet engraftment combined with persistent pulmonary infection after haploidentical allogeneic hematopoietic stem cell transplantation: a case report

**DOI:** 10.3389/fmed.2026.1814549

**Published:** 2026-04-24

**Authors:** Ying He, Yaxin Xiong, Juanjuan Zhang, Xinyu Gan, Fangyi Fan, Hai Yi

**Affiliations:** 1 Department of Hematology, Chinese People’s Liberation Army The General Hospital of Western Theater Command, Chengdu, Sichuan, China; 2 Sichuan Clinical Research Center for Hematological Disease, Chengdu, Sichuan, China; 3 Branch of National Clinical Research Center for Hematological Disease, Chengdu, Sichuan, China; 4 School of Public Health, North Sichuan Medical College, Nanchong, Sichuan, China; 5 Department of Orthopedics, Chinese People’s Liberation Army The General Hospital of Western Theater Command, Chengdu, Sichuan, China; 6 Department of Blood transfusion, Chinese People’s Liberation Army The General Hospital of Western Theater Command, Chengdu, Sichuan, China

**Keywords:** haplo-HSCT, poor graft function (PGF), poor platelet engraftment, pulmonary infection, rhizopus microsporus

## Abstract

Poor graft function (PGF) with persistent thrombocytopenia is a serious post-transplant complication linked to increased mortality. Active infections can worsen outcomes. We report a case of secondary poor platelet engraftment with refractory pulmonary infection after haploidentical HSCT. A 52-year-old woman developed Rhizopus microsporus infection on day +1. Neutrophil engraftment occurred by day +13, but platelet engraftment was delayed until day +40. She later developed *Pseudomonas aeruginosa* septic shock (day +106) and multidrug-resistant *Klebsiella pneumoniae* superinfection (day +190). Thrombopoietin receptor agonists showed minimal response, and she died 6 months post-transplant. This case highlights ultra-early mucormycosis, TPO-RA failure during active infection, and a fungal-bacterial cascade, underscoring the need for infection control before PGF-directed therapy.

## Introduction

Poor graft function (PGF), particularly persistent thrombocytopenia, is a serious complication following allogeneic hematopoietic stem cell transplantation (allo-HSCT) and is strongly associated with increased non-relapse mortality (1–4). Concurrent active infections can create a vicious cycle, further compromising engraftment and response to therapy (3). We herein report a challenging case of secondary poor platelet engraftment intertwined with refractory pulmonary infection after haploidentical HSCT.

## Case Report

A 52-year-old woman was diagnosed with high-risk acute mixed-phenotype leukemia with a complex chromosomal karyotype (46, XX,add(1), (q32)[4]/46, idem,de1(3)(p21), add(7)(q32), −22, + mar[7]/46, XX[9]). Post one course of daunorubicin hydrochloride 20 mg once daily on days 1–3, cytarabine 0.05 g every 12 h on days 1–5, vindesine sulfate 1 mg once weekly, and prednisone acetate 30 mg twice daily on days 1–14 and 20 mg on days 15–28 (DOAP), and one course of idarubicin 10 mg on days 1–3, fludarabine 40 mg on days 1–5, and cytarabine 3 g on days 1–5 (IDA-FLAG) chemotherapy, complete remission (CR) was achieved. Subsequently, one course of IDA-FLAG consolidation was administered. In April 2023, based on the clinical indication and the patient’s strong preference, an emergency haploidentical allo-HSCT procedure was performed due to the presence of two high-risk factors: complex chromosome karyotype and suspected leukemia infiltration in the right lobe of the liver on PET-CT. Transplantation pre-treatment regimen: cytarabine/decitabine/busulfan/fludarabine/etoposide/antithymocyte globulin (ATG). Ursodeoxycholic acid was given to prevent VOD. The prophylactic regimen for graft-versus-host disease (GVHD) was cyclosporine/mycophenolate mofetil/short-course methotrexate. Voriconazole was used for antifungal prophylaxis, and letermovir was given in sequence with ganciclovir for viral prophylaxis. Sulfamethoxazole-trimethoprim was used to prevent *Pneumocystis jirovecii* pneumonia (PJP). On day 1, 24 April 2023, 139 mL of donor peripheral blood stem cells were infused (TNC 11.11 × 10^8^/kg, CD34^+^ 6.2 × 10^6^/kg, MNC 8 × 10^8^/kg, CD3 1.17 × 10^8^/kg, weight 55 kg). On day 2, umbilical cord blood stem cells were infused.

**Figure 1:**
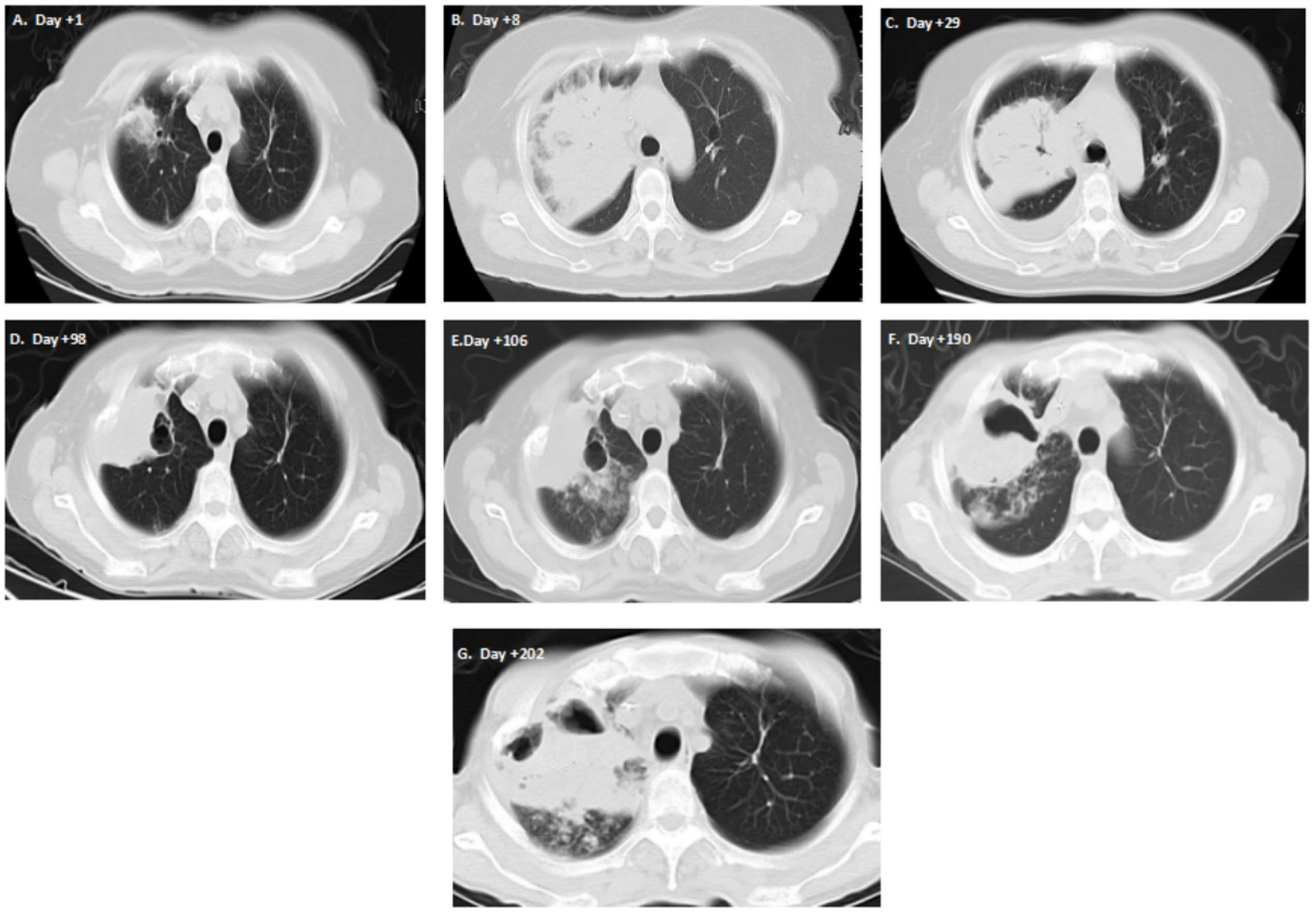
Serial chest CT images show pulmonary infection progression. **(A)** Day +1: Mass-like consolidation in the right upper lobe. **(B)** Day +8: Significant lesion progression. **(C)** Day +29: Cavitation development. **(D)** Day +98: Slight cavity enlargement. **(E)** Day +106: Lesion expansion during septic shock. **(F)** Day +190: Further progression with multidrug-resistant infection. **(G)** Final scan: Extensive consolidation.

On day +1, the post-transplant course was complicated by febrile (38.0 °C) neutropenia. Chest CT revealed a mass-like consolidation in the right upper lobe (Figure 1A), and blood next-generation sequencing (NGS) detected *Rhizopus microsporus* (sequence number 59), leading to a diagnosis of invasive fungal pneumonia and fungemia. After administering imipenem and teicoplanin for antibacterial treatment, isavuconazole orally, and amphotericin B inhalation for antifungal therapy, the patient remained hyperpyrexia. By day +8, the pulmonary lesion had significantly progressed (Figure 1B), and NGS again confirmed *Rhizopus microsporus* (sequence count 539). After adding amphotericin B cholesteryl, the peak body temperature gradually decreased. Following sore throat and vomiting, imipenem was replaced with meropenem, and symptoms improved. While neutrophil engraftment occurred on day +13 (absolute neutrophil count >0.5 × 10^9^/L for three consecutive days), platelet engraftment was severely delayed (<20 × 10^9^/L) and occurred on day +40. On day +14, whole blood chimerism showed 99.25% donor cells, and T-cell chimerism was 98.69% donor. Bone marrow smear and flow cytometry confirmed CR. The patient became afebrile. Treatment was adjusted to amphotericin B cholesteryl for antifungal therapy and cefoperazone-sulbactam for antibacterial coverage. Later, due to persistent hypokalemia, isavuconazole was resumed.

Despite negative follow-up NGS for *Rhizopus* on day +29, chest CT showed slight lesion shrinkage with new cavity formation (Figure 1C). Serial bone marrow examinations consistently demonstrated CR.

Antifungal therapy was switched to oral posaconazole (300 mg daily). On day +73, azacitidine 50 mg was administered on days 1–3 for relapse prevention. This resulted in chemotherapy-induced bone marrow suppression: platelet count dropped to 11 × 10^9^/L, recovering to 63 × 10^9^/L after transfusion. Subsequent imaging on day +98 showed slight cavity enlargement (Figure 1D).

One week later, on day +106, the patient developed a high fever (39.0 °C) accompanied by coughing, hemoptysis (≈25 mL/24 h), and sudden hypotension (75/40 mmHg). Chest CT revealed a lesion expansion (Figure 1E). Blood and sputum cultures grow *Pseudomonas aeruginosa*, indicating septic shock. Laboratory findings showed: WBC 0.24 × 10^9^/L, hemoglobin 70 g/L, platelet 7 × 10^9^/L, neutrophil 0.16 × 10^9^/L, CRP 86.24 mg/L, and PCT 0.41 ng/mL. Both CRP and PCT were elevated from baseline. Despite severe granulocytopenia with recurrent fever, meropenem and cefoperazone-sulbactam were added sequentially for antibacterial coverage, amphotericin B cholesteryl for antifungal therapy, sulfamethoxazole-trimethoprim for PJP prophylaxis, and valacyclovir for viral prophylaxis. Cyclosporine and tacrolimus were used sequentially for GVHD prevention due to a decreasing endogenous creatinine clearance rate (peak 45.6 mL/min).

**Figure 2:**
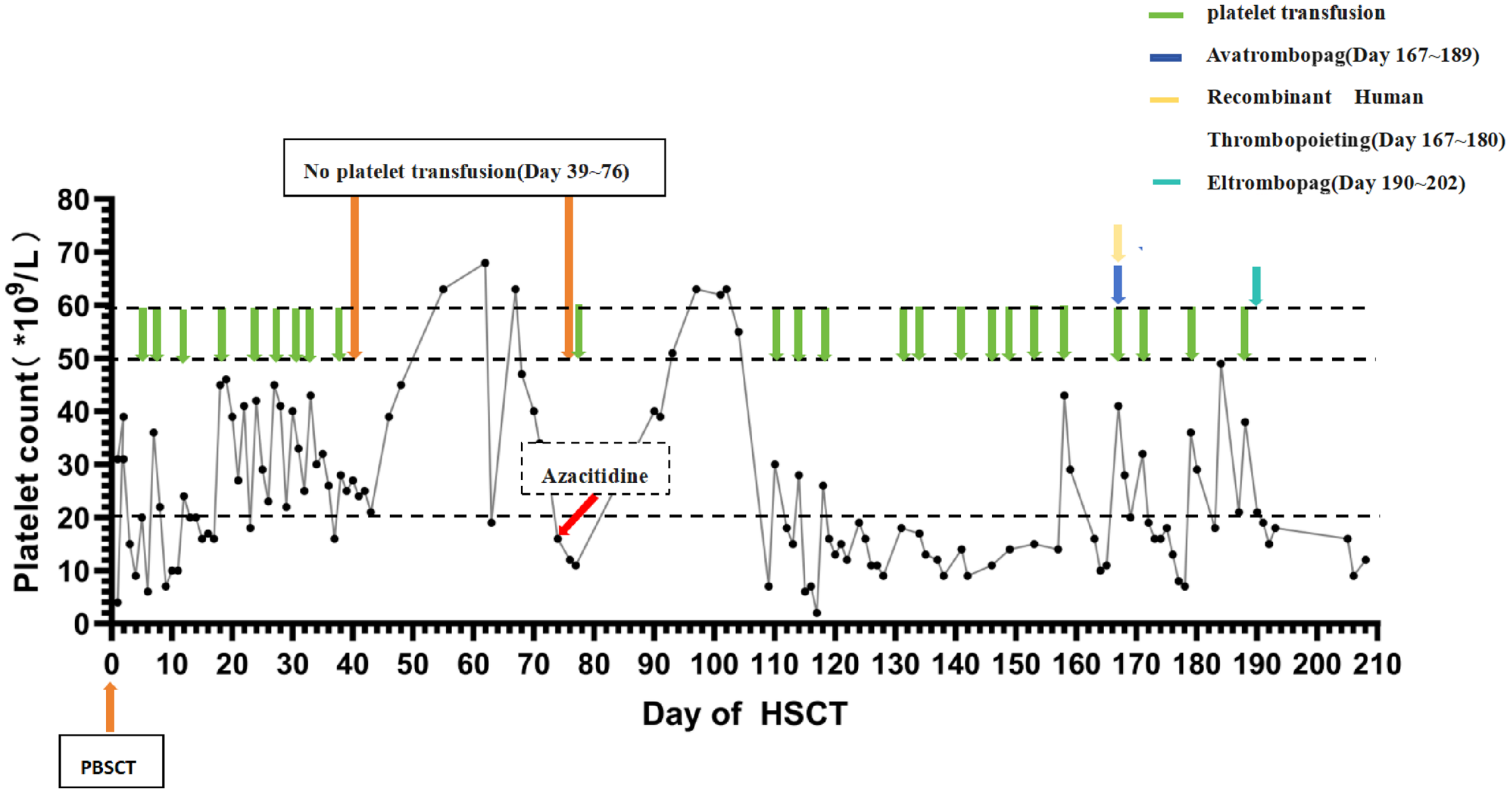
Trajectory of platelet alterations post-stem cell transplantation.

Although antimicrobial therapies were intensified, her condition deteriorated. Ferritin level was 3,827 ng/mL. Based on blood count findings, absence of hemophagocytosis on follow-up bone marrow examinations, normal soluble IL-2 receptor levels (<0.01 pg./mL), preserved NK cell function, and lack of progressive splenomegaly or hypertriglyceridemia, the observed hyperferritinemia in this patient was most likely attributed to systemic inflammation secondary to infection, rather than hemophagocytic lymphohistiocytosis (HLH) (5). HLH was therefore excluded, and iron chelation therapy with deferasirox (20 mg/kg/d) was continued. Whole blood and T-cell chimerism remained fully donor (>95%). More than 5-month post-transplant, platelet count remained at 16 × 10^9^/L (Figure 2), meeting criteria for secondary PGF (1, 2). Sequential treatment with avatrombopag (40 mg/day × 9 days switched to 60 mg/day × 2 weeks because of poor effect), recombinant human thrombopoietin (15,000 U/d × 2 weeks) and eltrombopag (50 mg/day×10 days) the effect was unsatisfactory, switched to 75 mg/d × 1 week), yielded a minimal response (peak platelet 18 × 10^9^/L without platelet transfusion).

On day +190, sputum cultures grew multidrug-resistant *Klebsiella pneumoniae* (carbapenem-resistant, sensitive only to tigecycline and colistin). Pulmonary infection progressed further (Figures 1F,G). Given the persistent thrombocytopenia, platelet transfusion was ineffective, and surgical repair of the pulmonary cavities could not be performed. Eventually, the patient succumbed to septic shock 6-month post-transplantation.

## Discussion

This case of secondary PGF with refractory *Rhizopus microsporus* infection after haploidentical HSCT demonstrates several unique features with important clinical implications.

The exceptionally early onset (day +1) and rapid progression of invasive mucormycosis are rare. Despite aggressive antifungal therapy with multiple agents (isavuconazole, amphotericin B cholesteryl, and posaconazole), the infection followed a relapsing course with cavitation and subsequent bacterial superinfections. This highlights the profound immunodeficiency in the early post-transplant period and the limitations of current antifungal strategies. Recent studies suggest that combination antifungal therapy and early surgical intervention may improve outcomes in mucormycosis, though data in transplant recipients remain limited (5).

This case illustrates the complex bidirectional relationship between PGF and infection. The patient met strict criteria for secondary PGF: persistent platelets <20 × 10^9^/L at 5 months, complete donor chimerism (>95%), and absence of relapse, severe GVHD, or myelosuppressive medications. The pathogenesis likely involves multiple factors: pre-transplant chemotherapy-induced damage, early fungal infection triggering systemic inflammation, and subsequent bone marrow microenvironment injury (3). Pro-inflammatory cytokines can directly suppress megakaryopoiesis and damage stromal cells supporting hematopoiesis (6).

The lack of response to sequential TPO-RA therapy (avatrombopag, recombinant human thrombopoietin, and eltrombopag) underscores that in the setting of active infection, stimulating platelet production is often ineffective. Recent literature emphasizes that infection control is paramount before attempting PGF-directed therapies (7). A large multicenter study found that active infection at the time of CD34 + -selected stem cell boost (SCB) was associated with significantly lower response rates (20% vs. 77%) and worse survival (8). Our patients repeatedly missed the window for SCB due to persistent or recurrent infection.

Current management strategies for PGF with concurrent infection emphasize a multimodal approach (1, 2, 4). First, aggressive pathogen identification using molecular methods (NGS) and tailored antimicrobial therapy are essential. Second, immunomodulation to reduce inflammatory damage while preserving anti-infective immunity represents a therapeutic frontier. Third, when infection is controlled, SCB should be considered early (before day +120) as it offers the highest likelihood of multilineage recovery (8, 9). Fourth, TPO-RAs may have adjunctive roles but should not delay definitive interventions when infection precludes response.

The prognostic implications of this case are sobering. The combination of PGF and refractory infection creates a self-perpetuating cycle: inflammation impairs hematopoiesis, cytopenia compromises pathogen clearance, and tissue damage (e.g., hemoptysis) provides portals for superinfection. Breaking this cycle requires integrated, multidisciplinary care with attention to both hematopoietic support and infection control.

In conclusion, this case illustrates that poor platelet engraftment coupled with persistent pulmonary infection forms a particularly ominous combination after haploidentical HSCT. It necessitates an integrated management strategy emphasizing early pathogen identification, aggressive antimicrobial therapy, and timely consideration of SCB once infection is controlled. Future research should focus on strategies to modulate inflammation while preserving immune function, and on optimizing the timing and sequence of PGF-directed therapies during active infection.

## Data Availability

The original contributions presented in the study are included in the article/supplementary material, further inquiries can be directed to the corresponding authors.
